# Healthcare Workers' Perspectives on the Barriers to Providing HIV Services to Children in Sub-Saharan Africa

**DOI:** 10.1155/2019/8056382

**Published:** 2019-03-03

**Authors:** Chipo Mutambo, Khumbulani Hlongwana

**Affiliations:** Public Health Medicine, School of Nursing and Public Health, University of KwaZulu-Natal, Durban, South Africa

## Abstract

**Background:**

In order to accelerate the HIV response to meet the UNAIDS 90-90-90 indicators for children, healthcare workers need to lead a scale-up of HIV services in primary healthcare settings. Such a scale-up will require investigation into existing barriers that prevent healthcare workers from effectively providing those services to children. Furthermore, if the identified barriers are not well understood, designing context-specific and effective public health response programmes may prove difficult.

**Objective:**

This study reviews the current literature pertaining to healthcare workers' perspectives on the barriers to providing HIV services to children in the primary care setting in Sub-Saharan Africa.

**Methods:**

English articles published between 2010 and April 2018 were searched in electronic databases including Sabinet, MEDLINE, PubMed, and Google Scholar. Key search words used during the search were “healthcare workers' perspectives” and “barriers to providing HIV testing to children” OR “barriers to ART adherence AND children” and “barriers to HIV disclosure AND children.”* Results*. There are various barriers to provider-initiated counselling and testing (PICT) of children and disclosure of HIV status to children, including the following: lack of child-friendly infrastructure at clinics; lack of consensus on legal age of consent for both HIV testing and disclosure; healthcare worker unfamiliarity with HIV testing and disclosure guidelines; lack of training in child psychology; and confusion around the healthcare worker's role, which most believed was only to provide health education and clinical services and to correct false information, but not to participate in disclosure. Additionally, primary caregivers were reported to be a barrier to care and treatment of children as they continue to refuse HIV testing for their children and delay disclosure.

**Conclusion:**

Training, mentoring, and providing healthcare workers with guidelines on how to provide child-focused HIV care have the potential to address the majority of the barriers to the provision of child-friendly HIV services to children. However, the need to educate primary caregivers on the importance of testing children and disclosing to them is equally important.

## 1. Introduction

Sub-Saharan Africa (SSA) is home to 12% of the global population, yet it bears 71% (6.8 million) of the global burden of HIV [1, 2]. Sub-Saharan Africa is also home to 90% of the global burden of HIV-infected children aged <15 years [3]. While SSA's 33% decline in new infections among children aged 0-14 years achieved in 2016 is commendable, the HIV incidence rates remain unacceptably high in this age group [4]. Reduction in new infections has been attributed to improved antiretroviral treatment (ART) and access thereto and scale-up of effective prevention of mother-to-child transmission (PMTCT) programmes dubbed a significant public health success [2, 5]. However, despite the substantial impact of PMTCT in reducing child morbidity and mortality, immense challenges remain in the delivery of HIV services for children in low resource settings. One of the biggest challenges is that many HIV-infected pregnant women still do not access antenatal care and therefore miss out on the health facility-based PMTCT interventions [6]. After pregnancy, the opportunities for early infant diagnosis (EID) of HIV-exposed children are limited, and many children remain undiagnosed in their infancy. They often only present after they have developed advanced AIDS-defining illnesses, resulting in poor prognosis even after being given ART [6].

The failure of EID interventions, which should be done during infancy, undercut the value of implementing PMTCT if exposed children continue to be missed by the healthcare system. The South African Prevention of Mother-to-Child Transmission Evaluation (SAPMTCTE) study found that, of the 2856 HIV-exposed infants attending facilities that reported providing EID, 62% had had a known HIV status documented on the child-health card or they were waiting for the 6-week immunisation. The remaining 38% of HIV-exposed children had no HIV status documented on their road to health cards, nor were their mothers intending to request EID services at the 6-week immunisation visit, which potentially puts them at risk of being missed opportunities for EID [18]. Additionally, studies conducted in Zimbabwe alluded to the existence of undiagnosed “slow progressors” presenting with advanced HIV disease at health facilities in late childhood and bringing to light an unanticipated emergent epidemic of older survivors of MTCT [19, 20]. These studies also linked the existence of an emergent epidemic in older children to the failure of public health to measure and directly observe the survival patterns of slow progressors [19].

In Sub-Saharan Africa, finding children of the survivors of MTCT remains a challenge as the opportunities for reentry into mainstream HIV care and treatment programmes are limited [6]. In addition, existing HIV testing and counselling programmes seldom focus on children older than 18 months of PMTCT, despite the existence of provider-initiated counselling and testing (PICT) guidelines. These guidelines encourage healthcare workers to proactively offer HIV testing and counselling to all primary caregivers, regardless of their reason for visiting the facility. Some of the barriers to implementing PICT include healthcare worker shortages, healthcare worker belief that children who do not have any symptoms need not be tested [16], and the unwillingness of the primary caregivers of exposed children to consent to HIV testing for their children [12]. In addition, studies have attributed primary caregivers' unwillingness to consent to self-stigma, fear of being blamed by the child, and fear of traumatising the child [12, 21].

After HIV diagnosis and HIV treatment initiation of a child, new challenges arise. Studies have shown that adherence to the HIV treatment regimen is problematic for children below the age of 14 years [22, 23]. This is because children are dependent on parents and other family members for access and support to correctly take their medication [24]. Studies have found that a primary caregiver's willingness to collect medication for the child and administer the medication is a determinant of the child's adherence to treatment [24, 25]. Moreover, studies have also reported that children of school-going age default because their lifestyles prevent them from going to the health facility for their monthly clinical assessment and drug pickup [26–28]. These behaviours put them at high risk of defaulting, as paediatric formulations require constant review and adjustments in accordance with fluctuations in weight. Some research blames children's nonadherence to ART on the lack of child-friendly palatable paediatric ART formulations resulting in children's refusal to take the medicine [29]. Research has also linked nonadherence to ART among children to the child's lack of knowledge of their HIV-positive status (child not disclosed to), lack of understanding of the consequences of defaulting, and inadequate knowledge about HIV and how the ARVs work [7]. Literature suggests that adherence to HIV treatment is more effective if the child has been disclosed to, is aware of his/her HIV-positive status, and understands the importance of adhering to the treatment regimen [11]. However, the challenge in most Sub-Saharan countries is that, by law, the decision to test the child and to disclose HIV status to a child under the age of 18 years remains the responsibility of the primary caregiver, and the healthcare worker can only support the process [23].

There is evidence suggesting that healthcare workers fail to provide children with HIV services because they lack adequate knowledge and skills to approach children and their caregivers [12, 21]. This is exacerbated by healthcare workers' lack of training on existing guidelines for providing child-friendly HIV testing services and disclosure counselling [8, 12, 15]. A study conducted in Ghana found that healthcare workers were unsure of the language or approach to use, particularly when providing counselling and health education during HIV testing and counselling services (HTS), and whether or not to provide these to the child or only discuss with the primary caregiver [21]. This is a huge concern as the WHO HIV testing and counselling guidelines [30] recommend that the child is provided with appropriate pre- and posttest counselling, adherence counselling, and basic HIV education as standard HIV services.

Providing children with HIV testing, adherence, and disclosure services after PMTCT remains a huge challenge in Sub-Saharan Africa. Therefore, it is imperative to ensure that the barriers preventing healthcare workers from providing these services to children in Sub-Saharan Africa are examined. This will be the first step towards developing and implementing viable solutions. The study focuses on “healthcare worker perspectives” because healthcare workers are the face of healthcare in the delivery of HIV services to children and their primary caregivers.

## 2. Research Question

What are the healthcare worker's perspectives on barriers which prevent them from providing quality HIV testing and adherence and disclosure services to children in Sub-Saharan Africa?

## 3. Methods

The systematic review process followed these four steps: (i) literature search, (ii) relevance screening, (iii) quality assessment and data extraction, and (iv) data analysis and summation.

## 4. Literature Search

### 4.1. Search Strategy

An electronic search of English articles published between 1 January 2000 and 30 April 2018 was done to retrieve relevant articles from electronic databases including MEDLINE, PubMed, and Google Scholar. In the context of this study, HIV services for children include HIV counselling and testing (HCT), ART initiation, treatment adherence, and disclosure counselling and support. The term “children” in this study refers to children between the ages of 0-14 years. Key search words used during the search were “healthcare provider OR health provider OR health worker OR healthcare worker OR nurse OR provider OR lay counsellor OR community health provider AND perspectives AND barriers AND HIV testing OR HIV disclosure OR ART adherence OR HIV medication AND children.” To increase the sensitivity of the search words, we used keywords and MeSH (Medical Subject Headings) terms and reviewed references from studies identified earlier.

## 5. Relevance Screening

### 5.1. Inclusion Criteria

The PRISMA guidelines were used to guide the study and standardise the review process. Titles of peer-reviewed articles were included only if they met the following criteria: they needed to be conducted in Sub-Saharan Africa, published in English, and published between 1 January 2010 and 30 April 2018 and have clear objectives and research methodology. Studies were included if they reported on perspectives/barriers/challenges of healthcare providers, healthcare workers, nurses, or community workers with regard to HIV testing of children OR HIV adherence support services for children OR HIV disclosure services for children. To manage the references, exclude duplicates, and find full texts, Endnote Reference Manager X8 software was used.

### 5.2. Exclusion Criteria

Duplicates, review papers, irrelevant objectives, and studies conducted from outside of Sub-Saharan Africa or outside the specified study period were excluded. No quality assessment of the studies was done. This study used existing published literature; therefore, no ethical approval was sought.

## 6. Quality Assessment and Data Extraction

The selection of papers for inclusion in this systematic review involved a three-step process as follows.

**Step 1 .**

A database search using keywords and title screening returned 503 articles; 39 additional records were identified through reference lists and public health organisations' websites. After applying the exclusion criteria and identifying duplicates records a total of 225 records were excluded.

**Step 2 .**

Screening titles and abstracts identified 29 records for full-text review after exclusion of 288 papers which either were not conducted in Sub-Saharan countries or reported on perspectives that were not from healthcare workers.

**Step 3 .**

Full article screening resulted in the further exclusion of 9 articles which did not present clear results or had insufficient information or whose full text was unavailable. This resulted in only 11 records being assessed for quality using the Mixed Methods Appraisal Tool (MMAT) version 2018 checklist, which is designed for appraising the quality of systematic reviews of mixed studies reviews.

### 6.1. The Process of Extracting the Data from Studies

**Figure 1:**
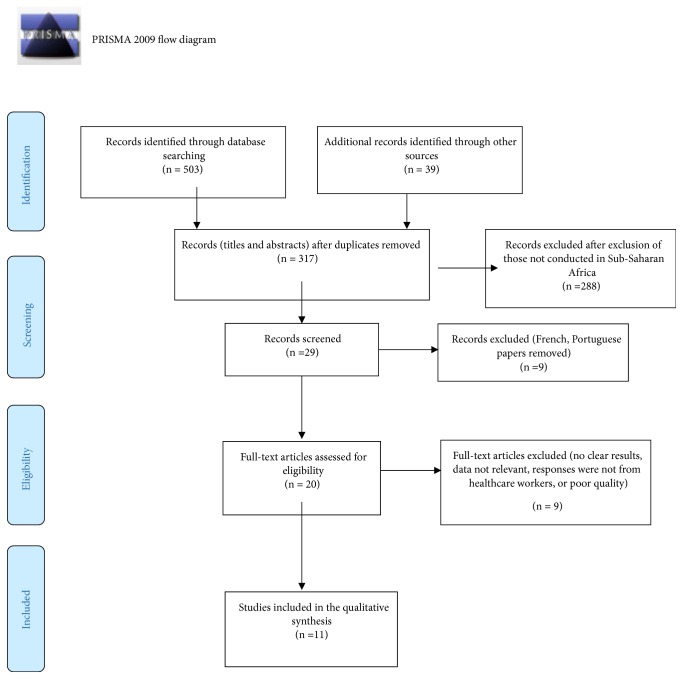
PRISMA flow diagram.

Two reviewers (CM and DH) were responsible for the choice of articles and data extraction from the studies that passed the quality assessment. When the decision was unclear, the two reviewers both conducted a reassessment and held discussions aimed at reaching a consensus. To extract the required information from each article, a customised data collection tool (see Table 1) with the titles title, HIV focus area, study objective, location, population, sample size, and study design and methods of data collection was used. To map the process of searching articles included in the review, we used the PRISMA diagram tool [31] (Figure 1). A total of 20 journal articles met the inclusion criteria and the quality criteria but 9 were excluded because the authors could not access the full-text articles or they did not have full results. Finally, 11 papers were selected for the final analysis. Following data extraction, manually conducted content analysis was done by CM and DH to identify key categories and report on key findings. A summary of the results from the analysis is presented in Table 2.

## 7. Ethical Considerations

This literature review was part of a larger doctoral study approved by the University of KwaZulu-Natal Biomedical Research Ethics Committee (BREC) (Ref. No. BE298/18) and the KwaZulu-Natal Department of Health (Ref. No. KZ_201809_011).

## 8. Results and Discussion

### 8.1. Included Studies

Only studies from Sub-Saharan Africa (see Table 1) were included in this study and of these, five were from South Africa [7, 8, 13, 14, 16], two were from Zimbabwe [11, 12], and one was from Namibia [9], Kenya [10], and Ethiopia [17] each. About 82% (9/11) of these studies were qualitative, one was a cross-sectional study, and the other used mixed methods.

Following content analysis, the barriers preventing the provision of quality HIV services were categorised as follows: healthcare worker-related barriers, primary caregiver barriers, and system-related barriers (Table 2).

### 8.2. Healthcare Worker-Related Barriers

In all 11 studies reviewed, healthcare workers were reported to lack formal training on paediatric HIV, thus making it difficult for them to confidently provide HIV services to children [7–17]. With regard to providing children with HIV counselling and testing, healthcare workers cited that they had inadequate knowledge around HIV and the law, and some were concerned that they would be legally liable if they provided tests and disclosed to children as recommended by the guidelines [12, 13, 16]. In addition, healthcare workers complained about the lack of child-friendly job aides which they felt were essential in the process of effectively communicating with children through using age-appropriate language. Providing PICT was also said to be difficult as most children are not accompanied to the health facilities by their legal guardians, and healthcare workers are forced not to test them [12]. In most studies, healthcare workers were aware that it was not their responsibility to disclose to children, but rather to support the primary caregiver and the child to make the process of disclosure smoother and to attend to questions that may arise [7, 8, 13]. However, some healthcare workers reported having disclosed to children [13]. A study in Zimbabwe noted that there were concerns around testing unaccompanied children as well as vulnerable children in bad circumstances, as some felt that a positive test would put the child at risk of being maltreated and potentially even abandoned [12]. Finally, two studies reported that healthcare workers faced time constraints when providing comprehensive HIV counselling and testing services to children and providing disclosure services [8, 13].

### 8.3. Primary Caregiver-Related Barriers

Primary caregivers are often unwilling to consent to HIV counselling and testing of their children, fearing that such action will lead to revealing their previous HIV secrecy to children [7, 8, 11, 13]. In most studies, stigma, fear of blame, and the fear of a child's negative psychological reaction were cited by primary caregivers as their main reasons for refusing to have their children tested for HIV [7, 8, 11, 13]. On the other hand, healthcare workers attributed primary caregivers' refusal to have their children tested for HIV to their lack of awareness about the benefits of disclosing to children. The lack of training of healthcare workers on how to effectively provide HIV testing and disclosure support services makes it difficult for primary caregivers to make informed choices regarding their children [7, 8, 11, 13]. Moreover, primary caregivers were reported to be the main barriers to adherence for children as some were not committed to collecting their child's medication on time and in most cases picked up medication without the children [17].

### 8.4. System-Related Barriers

Healthcare workers noted that, despite the fact that the vulnerability of children is well known, the healthcare system does not provide adequate, holistic, and child-centred services to benefit children [12]. Healthcare workers further noted that there were no formal recommendations or guidelines to assist healthcare workers to provide appropriate child-focused HIV services to children and their primary caregivers [8, 12, 15]. In addition, all studies highlighted the lack of formal institutional training of child-focused care for the provision of HIV services to children [7–17]. In a study looking into the barriers to providing HIV services via IMCI, one healthcare worker reportedly feared writing about “Suspected HIV” in the child's clinical stationery as she feared the risk of litigation [16]. Regarding children's nonadherence to ART, healthcare workers highlighted that the medication available to patients was unpleasant and not child-friendly, thus contributing to children's refusal to take their medication and negatively impacting on adherence [17].

### 8.5. Discussion

This review identified key barriers impeding the successful provision of HIV services to children, mainly from the perspectives of healthcare workers. Findings from this review suggest that healthcare workers in Sub-Saharan Africa lack the knowledge, skills, and tools to enable them to provide comprehensive HIV services to children [7–17]. Furthermore, healthcare workers recognise the value of children in healthcare and the importance of tailoring their care to make it age-appropriate. Studies conducted in Namibia highlighted that more children were disclosed to in health facilities where trained healthcare workers and child-friendly storybooks were available [9]. Similar studies also suggest that the age and maturity of the child is a key factor to consider when providing HIV services [7, 8, 11, 13]. Another study conducted in Zimbabwe highlighted that healthcare workers often referred newly disclosed-to adolescents to peer support groups, in order to aid their transition and ensure that they have age-appropriate psychosocial care provided by peers in the same situation [12].

Stigma and discrimination associated with HIV remain rife in all Sub-Saharan African countries, undermining all the potential benefits of early infant diagnosis, early ART initiation, disclosure, and adherence to treatment [7–17]. The review also makes it clear that primary caregivers play a crucial role in the HIV care of their children, and therefore HIV interventions for children should also be family-focused and inclusive of both the child and his/her primary caregiver. Moreover, such family-focused interventions have the potential to create healthy and open relationships in the family which are beneficial to HIV-positive children's adherence and retention in care, as they ensure that a child is provided with necessary psychosocial support. Moreover, the study showed a consensus when it comes to the nature of disclosure with all studies acknowledging that it is a complex issue which is incrementally governed by many factors. These factors include the age and maturity of the child, the self-efficacy, confidence, and willingness of the primary caregiver to disclose to the child, and the persuasive skill of a healthcare worker to convince the primary caregiver and to facilitate the process as needed [7–17].

This review also showed that the health systems in Sub-Saharan Africa are struggling to adequately equip healthcare workers with tools to enable them to effectively conduct their work. Unavailable tools included paediatric HIV guidelines around HIV testing and disclosure, child-friendly job aides, and a child-friendly environment in health facilities [7–17]. In addition, one study looking at the barriers to children's adherence to treatment noted that the lack of child-friendly paediatric formulations makes it difficult for children to adhere to treatment [17]. Existing ART medication is unpleasant, must be taken daily, and is lifelong with children, then, at risk of developing treatment fatigue and treatment default. Finally, Sub-Saharan Africa continues to be plagued by human resource shortages, shortage of HIV testing kits, and drug stock-outs, which continue to threaten the public health of its people and the realisation of the 90-90-90 treatment goals [10].

### 8.6. Limitations of the Study

One of the limitations of this study was that of the exclusion of literature from Francophone Africa which could have potentially improved the diversity of the body of literature reviewed by the authors and reduced publication bias. In addition, only 11 articles were identified for review in this publication due to various factors stated in the methodology; hence this paper may not be reflective of all healthcare systems in Sub-Saharan Africa, in so far as the provision of HIV services to children is concerned.

### 8.7. Implications of the Study

In Sub-Saharan Africa, HIV interventions have mostly been focused on children in the PMTCT age group, thereby neglecting older children and adolescents [9]. Nevertheless, a new problem has surfaced through these PMTCT programmes, including the survival of HIV-positive children into late childhood, adolescence, and adulthood. These developments make disclosure a pertinent issue, especially in Sub-Saharan Africa, a region which is home to 90% of the world's HIV burden [32]. In addition, studies have highlighted the inadequacies of PMTCT, suggesting that some HIV-positive children remain undiagnosed and efforts should be made to equip providers with skills to actively offer HIV tests and initiate these children on ART. It is, therefore, imperative that decision-makers take all necessary steps to equip healthcare workers with training, mentorship, and job aides to be able to improve the quality of HIV services and ensure that newly diagnosed children access care and have a positive healthcare experience and remain in care and adherent to their treatment. Finally, decision-makers need to be informed about the barriers preventing their staff from providing the best possible care for children to enable them to allocate Sub-Saharan Africa's limited resources to the most pertinent problems.

## 9. Conclusions

This study reviewed healthcare workers' perspectives on the barriers preventing them from providing quality HIV testing, adherence, and disclosure services to children in Sub-Saharan Africa. Child-centred approaches should be adopted by healthcare providers to ensure that children receive holistic and age-appropriate care. Developing formal guidelines, training and mentoring healthcare workers on these child-focused approaches, developing child-friendly job aides, and creating child-friendly areas have a potential to marginally improve the quality of HIV services provided to children in low resource settings. This will, in turn, push Sub-Saharan Africa closer to achieving its 90-90-90 goals.

## Figures and Tables

**Table 1:** A summary of the studies identified.

Reference	Title	HIV focus	Study objective	Location	Population sample size	Study design and methods of data collection
		area				
[7]	Health care workers' perspectives about disclosure to HIV-infected children; cross-sectional survey of health facilities in Gauteng and Mpumalanga provinces, South Africa	Disclosure	To assess how disclosure to HIV-infected children is being implemented in public health facilities	South Africa	206, 140 (68.2%) were nurses, 44 (21.5%) were lay counsellors, and 4 (2%) were doctors	A cross-sectional survey using a semistructured questionnaire

[8]	Healthcare providers' perspectives on discussing HIV status with infected children	Disclosure	To investigate South African healthcare providers' perspectives on discussing HIV status with infected children	South Africa	40 providers, 11 doctors, 13 nurses, and 7 others (social workers and pharmacists)	Qualitative using structured interviews

[9]	“If I Take My Medicine, I Will Be Strong:” Evaluation of a Paediatric HIV Disclosure Intervention in Namibia	Disclosure	To evaluate healthcare worker and caregiver perspectives on the effectiveness of the intervention in increasing their capacity to engage in the disclosure process and improving paediatric patient adherence behaviour	Namibia	35 healthcare workers and 46 caregivers of HIV-positive children	Qualitative interviews using semistructured questionnaires

[10]	Using a Health Provider Insights to Inform Paediatric HIV Disclosure: A Qualitative Study and Practice Framework from Kenya	Disclosure	To determine processes, concerns, successes, beliefs, and experiences of providers surrounding paediatric HIV disclosure	Kenya	21 providers, 3–5 from each clinic; 2 clinicians, 5 clinical officers, 3 nurses, 3 nurse counsellors, 2 psychologists, 1 clinic assistant, and 1 peer educator	Qualitative interviews using semistructured questionnaires

[11]	HIV Status Disclosure to Perinatally-Infected Adolescents in Zimbabwe: A Qualitative Study of Adolescent and Healthcare Worker Perspectives	Disclosure	To examine healthcare worker and adolescent perceptions of the disclosure process in health facilities	Zimbabwe	31 (14 male, 17 female) prenatally infected adolescents aged 16–20, 15 (1 male, 14 female) healthcare workers	Qualitative interviews using semistructured questionnaires

[12]	Barriers to Provider Initiated Testing and Counselling for Children in a High HIV Prevalence Setting: A Mixed Methods Study	PICT	To investigate the provision and uptake of HIV provider-initiated counselling and testing (PICT) among children in primary healthcare settings and to explore healthcare worker perspectives on the provision of HIV testing to children	Zimbabwe	Children aged 6 to 15 years and 12 healthcare workers	Qualitative interviews using semistructured questionnaires

[13]	‘Are we allowed to disclose?': A Healthcare Team's Experiences of Talking with Children and Adolescents about their HIV status	Disclosure	To explore the perspectives and experiences of a healthcare team at a paediatric clinic in South Africa about disclosure to children	South Africa	23 healthcare providers	Qualitative study-focus group discussions

[14]	Factors influencing uptake of HIV care and treatment among children in South Africa-a qualitative study of caregivers and clinic staff	HIV care and treatment	To explore the perspectives of clinic staff and caregivers of children enrolled in HIV services on barriers and facilitators of children's uptake of HIV care	South Africa	21 caregivers of HIV-infected children attending these clinics, 21 clinic staff members, and three lead members of staff from affiliated care centres	Qualitative study-interviews using semistructured questionnaires

[15]	“Experiences with the disclosure of HIV-positive status to the infected child”: Perspectives of healthcare providers in Dar es Salaam, Tanzania	Disclosure	To explore experiences of healthcare providers in the disclosure of HIV-positive status to the infected child and factors influencing the process	Tanzania	20 healthcare providers	Qualitative interviews using semistructured questionnaires

[16]	Routine checks for HIV in children attending primary health care facilities in South Africa: Attitudes of nurses and child caregivers	HIV counselling and testing services	To describe the attitudes towards, and experiences of, implementation of routine checks for HIV in the context of IMCI implementation, from the perspective of both caregivers and nurses	South Africa	5 with Integrated Management of Childhood Illness- (IMCI-) trained nurses (3 in KwaZulu-Natal and 2 in Limpopo) and 5 with mothers and caregivers (3 in KwaZulu-Natal and 2 in Limpopo), 10 nurses in each district for each focus group	Qualitative study-focus group discussions

[17]	Barriers and facilitators to antiretroviral medication adherence among HIV infected paediatric patients in Ethiopia: A qualitative study	Antiretroviral therapy adherence	To explore the barriers and facilitators to Highly Active Antiretroviral Therapy (HAART) adherence among HIV-infected children	Ethiopia	12 caregivers of nonadherent children, 14 key informants, eight counsellors, and four physicians, including two paediatricians	Qualitative interviews using semistructured questionnaires

**Table 2:** A summary of the barriers to providing HIV testing, adherence, and disclosure services to children in Sub-Saharan Africa.

Themes	Key barriers	Studies identified
Healthcare worker-related barriers	(i) Healthcare workers lack formal training on child-friendly approaches to enable them to provide adequate HIV services to children	[7–17]
	(ii) Healthcare workers have inadequate knowledge and understanding around HIV and the law	[12, 13]
	(iii) Inadequate knowledge of providing children with HIV services	[14]
	(iv) Lack of child-friendly job aides to improve their communication with both children and their primary caregivers	[9, 11]
	(v) Inadequate buy-in by healthcare workers into provider-initiated counselling and testing (PICT) for children	[12, 16]
	(vi) Confusion on child and primary caregiver consent for HIV testing, counselling, and disclosure	[12, 13]
	(vii) Healthcare workers lack the confidence to provide HIV services to children as they are not trained	[9]
	(viii) Healthcare workers have concerns about the safety of providing provider-initiated counselling and testing (PICT) to children who are sometimes unaccompanied as it has legal implications	[9]
	(ix) Healthcare workers complained of facing time constraints when providing primary caregivers and their children with disclosure support	[8, 13]

Primary caregiver-related barriers	(i) Healthcare workers reported that stigma is still rife which prevents primary caregivers from disclosing to their children	[7, 8, 11, 13]
	(ii) Children do not pick up their own medication; instead, primary caregivers pick it up which makes it difficult for healthcare workers to effectively clinically monitor them	[17]
	(iii) Primary caregivers lack an understanding of the benefits of disclosure which makes them unwilling to agree to disclose to their children	[12, 13, 16]

System-related barriers	(i) No training courses for healthcare workers are available concerning the provision of child-focused HIV care	[12, 16]
	(ii) No recommendations and guidelines are available to guide healthcare workers concerning the provision of child-focused HIV services to children	[8, 12, 15]
	(iii) Staff shortages in facilities prevent healthcare workers from providing HIV services to children	[8]
	(iv) The Integrated Management of Childhood Illness (IMCI) programme does not provide adequate HIV information for healthcare workers to use when providing HIV support and care to children	[16]
	(v) The Integrated Management of Childhood Illness (IMCI) programme clinical stationery is a limitation to full service delivery as a recording of HIV suspects may expose healthcare workers to legal liability	[16]
	(vi) Healthcare workers cited the lack of child-friendly and palatable antiretroviral formulations as barriers to adherence	[17]
	(vii) Healthcare workers also cited HIV testing kits and kit stock-outs as barriers to testing children in primary healthcare settings	[9]
	(viii) Healthcare workers also cited the lack of child-friendly areas in health facilities as barriers to improving children's healthcare experiences	[14]

## Data Availability

No additional unpublished data are available.
